# 

**Published:** 1894-04-14

**Authors:** 


**Notice to Advertisers and Subscribers.**

All Advertisements, Orders for Copies of The Hospital, and Business Communications should be addressed to **The Publisher, NOT TO THE EDITOR**, The Hospital, 428, Strand, W.C.

The Cost of Subscription, post free, is as follows:—Per week, 2½d.; per quarter, 2s. 8d.; per half-year, 5s. 3d.; per annum, 10s. 6d. Foreign:—Per Annum, 12s. 8d.; payable, in all cases, in advance.

**Notices of Births, Deaths, and Marriages** are inserted in this column at a charge of 2s. 6d. for an announcement not exceeding four lines.

### NOTICE TO CORRESPONDENTS.

All MS., Letters, Books for Review, and other matters intended for the Editor should be addressed The Editor, The Lodge, Porchester Square, London, W.

The Editor cannot undertake to return rejected MS., even when accompanied by stamped directed envelope.

**"Burdett's Hospital Annual," 1894.**

Fifth year of publication. 900 pp. Boards, gilt lettering. A most complete guide to British, Colonial, Indian, and American Hospitals, Dispensaries, Nursing and Convalescent Institutions, and Asylums. Price 5s. Published by The Scientific Press (Limited), 428, Strand, W.C.

**NOTICE.—The Index for Vol. XIV. (ending Sept. 30th, 1893) is on sale at the Office of this Journal, price 2½d., post free.**

### SCRAPS AND GLEANINGS.

Miss Anne Taylor Fox has left a legacy of £50 to the funds of the Huntingdon County Hospital.

Miss Grace Rolleston, of Hyde Park Terrace, has bequeathed £1,000 to the Throat Hospital, Golden Square.

The battle of the sites continues unabated at East Grinstead regarding the erection of the proposed isolation hospital in that locality.

A donation of £75 from the Magdalene Charity has been acknowledged in the report of the Bean Site Convalescent Home, Hastings.

The Merthyr General Hospital boasts of no less a balance in hand than £537 at the end of the year under review at the general meeting.

Baron de Hirsch has sent, per George Herring, Esq., a donation of £500 towards the funds of the Metropolitan Hospital, Kingsland Road, N.E.

Eighteen hundred and sixty-one persons have been treated in the out-patient department of the Chesterfield Hospital and Dispensary during the la t year.

The Hospital Saturday collection made by the ladies of Wolverhampton for the General Hospital has resulted in £29 being added to the funds of the institution.

The report on the Carlisle Dispensary shows that 3,964 patients had sought relief from the institution during 1893. This number is the highest recorded in the books.

A deficiency of £43 at the beginning of the year on the general account of the Weston-super-Mare Hospital and Dispensary is reported to have grown to £138 at the end.

Mr. Walter Cunliffe has lately sent £21 to the Epsom Cottage Hospital authorities to be paid out in small annual subscriptions for patients to be sent to convalescent seaside homes.

Plans, specifications, and estimates are forthwith to be called for by the promoters of the new cottage hospital at Wells, which building will owe its existence to the late Dean Plumtree's legacy.

By the kindness of several members of the Ladies' Committee a number of indoor patients of the Liverpool Ladies' Charity and Lying-in Hospital have had a fortnight's stay at a cottage at Woolston.

The East London Hospital for Children, Shadwell, has received a donation of £100 from Mr. John Stefanovich Schilizzi and Mr. Paul Stepnovich Schilizzi, in memory of their brother, the late Demetrius Schilizzi.

The Committee of the Lunatic Hospital, Coppice Road, Nottingham, laid much stress at the recent annual meeting on the soundness of the financial condition of the establishment, and the high state of efficiency in which it had been maintained.

The report of the National Eye and Ear Infirmary, Dublin, pointed out that the proposed amalgamation of this hospital with the St. Mark's Ophthalmic Hospital had been deferred on account of the difficulty as to the name the amalgamated hospital should bear.

Thanks to the liberal and hearty response to an appeal in the summer made for wider help towards the Maidenhead Cottage Hospital, the income of the hospital has exceeded that of last year, and the committee now report the satisfactory available balance of £52.

As a slight acknowledgment of the munificent gifts of two benefactors to the Newark Hospital, two wards have been named after these two special patrons. The female ward has been named the "Ossington" Ward, and the male ward the "Branston" ward.

It is proposed to institute a house-to-house collection in April throughout the district of the Stamford, Rutland, and General Infirmary. Amongst the directions in which increased funds would be expended is the establishment and endowment of a children's ward.

The expenditure at the Newry Fever Hospital is stated as having been exceptionally heavy during the past twelve months. About £175 had unavoidably to be laid out in sanitary improvements. Patients to the number of 439 received treatment at the hospital during this time.

The finances of the building fund towards the erection of the new infirmary at Lancaster are being augmented in no inconsiderable manner. Mr. Williamson, M.P., has added to his previous benevolence by contributing £1,300, and Mr. T. Walker has forwarded £100 for the same object.

The Ladies' Committee of the Liverpool Hospital for Women has initiated a canvass in favour of providing a permanent annual income in support of six additional beds in the hospital. Mr. Edward Thompson and Mrs. Sulton Timmis have subscribed £500 each towards this object.

A Local Government Board inquiry was held recently at the Workhouse, Redhill, concerning the application of the Reigate Rural Sanitary Authority for the sanction of the Local Government Board to borrow a sum of £2,500 for the purpose of enlarging the Surrey County Hospital.

The Duke of Beaufort presided lately at a meeting inaugurated by supporters of the Tetbury Cottage Hospital, the immediate object of this meeting being the clearing off of a debt of £40 which exists on the hospital accounts, also with the endeavour to add an extra £100 a year to the permanent funds.

In response to a pressing appeal from the inhabitants of the Tilbury district, Mr. Passmore Edwards has offered to build a cottage hospital at a cost of £2,000. At present cases demanding hospital treatment have to be sent to Gravesend on the one side of the Thames, and to the London Hospital on the other.

The report of the Darlington Hospital Committee with reference to a new children's ward, shows that it is troubled as to the cost of such an addition to the expenses. This proposed addition to the hospital is designed to carry on the work of Miss Emma Pease's Hospital for Sick Children which is now to be given up.

The good work effected by the Home and Infirmary for Sick Children at Lower Sydenham has further extended its sphere by opening a convalescent home at Frinton. This new branch of usefulness, which means an increased expenditure of £200, was carried out in commemoration of the parent "Home" having attained its majority.

### Medical Vacancies and Appointments.

**Appointments.**

A. Byers, M.B., Ch.B.Vict., B.Sc.Lond., appointed House Physician to the Manchester Royal Infirmary. H. A. Caley, M.D.Lond., M.R.C.P., appointed Joint Medical Tutor to St. Mary's Hospital, Paddington. C. M. Chadwick, M.A., M.D., M.R.C.P., appointed Honorary Physician to the Leeds General Infirmary. W. K. Clayton, M.D.Brux., L.R.C.P., L.R.C.S. Edin., L.M., appointed Medical Officer of the Wakefield District of the Wakefield Union and Public Vaccinator of the No. 1 District. J. L. Dalby, M.R C.S., L.R.C.P., appointed Senior House Surgeon to the East Suffolk and Ipswich Hospital. R. Dudfield, M.A.Camb., M.B., D.P.H., L.R.C.P.Lond., M.R.C.S.Eng., appointed Medical Officer of Health for the Borough of Paddington. S. Edgerley, M.A., M.B., C.M.Edin., appointed Assistant Medical Officer to the Roxburgh District Asylum, Melrose, N.B. E. C. Edwards, M.B., C.M.Edin., appointed Junior House Surgeon to the East Suffolk and Ipswich Hospital. J. H. Fletcher, M.R.C.S., L.R.C.P., appointed House Surgeon to the Manchester Royal Infirmary. W. K. Fyffe, M.B., M.R.C.P., appointed Medical Registrar to St. George's Hospital. W. Hammond, L.R.C.P.Edin., M.R.C.S., appointed Medical Officer for the No. 6 District of the Liskeard Union. G. W. Johnstone, L.R.C.P.Edin., L.F.P.S.Glas., reappointed Medical Officer of Health to the Upholland Local Board.

**VACANCIES.**

**Resident Medical Officers.**

The following vacancies are announced this week, the amount of salary in each case being given after the name of the institution:

Bury Dispensary Hospital, Bury, Lancashire.—Senior House Surgeon, doubly qualified. Salary £100 per annum, with board, residence, and attendance. Applications to the Secretary, Mr. Henry Webb, by April 16th.

Derby County Asylum.—Clinical Assistant. Appointment for six months Board, lodging, and washing provided. Applications to the Medical Superintendent, County Asylum, Mickleover, Derby.

Flintshire Dispensary.—House Surgeon. Salary £120 per annum, with furnished house, rent and taxes free; also coal, light, water, and cleaning, or, in lieu thereof, £20 per annum. Knowledge of Welsh desirable. Applications to W. T. Cole, Secretary, Board-room, Bagillt Street, Holywell, by May 15th.

Hospital for Consumption and Diseases of the Chest, Brompton.—Resident House Physicians. Applications to the Medical Committee by April 19th.

Newcastle-on-Tyne Dispensary.—Resident Medical Officer. Salary £250 per annum, with furnished residence. Applications to the Honorary Secretary, R. W. Sisson, 13, Grey Street, Newcastle-on-Tyne, by April 20th.

Royal Victoria Hospital, Bournemouth.—House Surgeon and Secretary. Salary £100 per annum, with board. Applications to the Chairman of Committee by May 1st.

The Coppice, Nottingham.—Assistant Medical Officer; unmarried, and not more than 28 years of age. Salary £120 per annum, with furnished apartments, board, washing, and attendance. Applications to Dr. Tate at the Asylum by April 14th.

West Riding Asylum, Wadsley, near Sheffield.—Fifth Assistant Medical Officer. Salary £100 per annum, rising £10 annually up to £150, with board, &c. Applications to the Medical Superintendent by April 17th.

Wirrall Children's Hospital, Woodchurch Road, Birkenhead.—Resident House Surgeon. Salary £50 per annum, with board, lodging on the premises, and washing. Applications to P. W. Atkin, Honorary Secretary, 25, Lord Street, Liverpool, by April 24th.

**Non-Resident Medical Officers.**

Dental Hospital of London, Leicester Square. — Two Assistant Anæsthetists. Applications to J. Francis Pink, Secretary, by May 14th.

Dental Hospital of London and London School of Dental Surgery, Leicester Square.—Demonstrator. Honorarium £50 per annum. Applications to Morton Smale, Dean, by May 14th.

### The Hospital Advertiser.

**THE COPPICE, NOTTINGHAM.**

A GENTLEMAN is REQUIRED for the POST of ASSISTANT MEDICAL OFFICER at this Asylum for Private Patients.

Candidates must be duly qualified and registered, and unmarried.

Age not to exceed twenty-eight years.

Salary £120 per annum, with Furnished Apartments, Board, Washing, and Attendance.

Applications, with copies of testimonials, to be forwarded to Dr. Tate at the Asylum, by April 14th. (101)

**RESIDENT HOUSE PHYSICIANS.**

